# Efficacy of Emla (Eutectic Mixture of Local Anaesthetics) and Let (Lidocaine, Epinephrine, Tetracaine) for Topical Use in Wound Management for Children: A Systematic Review and Meta-Analysis

**DOI:** 10.7759/cureus.31447

**Published:** 2022-11-13

**Authors:** Hany A Zaki, Mohamed A Elarref, Haris Iftikhar, Nood Dhafi R Al-Marri, Maarij Masood, Mohamed Fayed, Mohamed Abdelgadir M Elgassim, Nabil A Shallik

**Affiliations:** 1 Emergency Medicine, Hamad Medical Corporation, Doha, QAT; 2 Anaesthesiology, Weill Cornell Medicine-Qatar, Doha, QAT; 3 Department of Anesthesiology, ICU and Perioperative Medicine, Hamad Medical Corporation, Doha, QAT; 4 Clinical Anaesthesiology, Weill Cornell Medicine-Qatar, Doha, QAT; 5 Anaesthesiology, Hamad Medical Corporation, Doha, QAT

**Keywords:** systematic review and meta analysis, children, surgical wound management, topical anesthetics, lidocaine-epinephrine-tetracaine gel, let, lidocaine-prilocaine gel, emla

## Abstract

Lacerations are common injuries managed by emergency department practitioners and are mostly witnessed in children. These lacerations usually require wound closure, which may result in one of the most unpleasant and painful childhood experiences. The pain can be minimized through topical anesthesia, such as a combination of lidocaine, epinephrine, and tetracaine (LET) and a eutectic mixture of local anesthetics (EMLA). The current study was carried out to demonstrate the efficacy of EMLA and LET in pediatric wound management.

A thorough literature search was carried out without any time limitation on five electronic databases, including PubMed, Medline, Web of Science, Embase, and Google Scholar. Relevant studies from these databases and their references were scoured for additional studies. Study quality appraisal and data analysis were conducted using Review Manager software (RevMan 5.4.1).

The literature search yielded 1651 articles of which only eight were eligible for inclusion in the present study. A meta-analysis of results from 3 studies showed that LET had a significant pain reduction than the control interventions (SMD: -0.46; 95% CI: -0.69, -0.23: p<0.0001). However, the pooled effect size of data from 3 studies showed EMLA had an insignificant difference with the control interventions (SMD: -0.79; 95% CI: -1.82, -0.24: p = 0.13). Similarly, no significant difference in the number of adverse reactions was reported in either EMLA (OR: 2.31; 95% CI: 0.67, 7.93; p = 0.18) or LET (OR: 0.99; 95% CI: 0.15, 6.50; p = 0.99)

Our study suggests that the topical application of EMLA and LET effectively offers pain-free wound management among pediatric patients. However, the interventions are subject to adverse reactions that should be considered when managing the wounds.

## Introduction and background

Lacerations are among the most common injuries managed by emergency department (ED) practitioners. It is estimated that in the United States alone, approximately 7 million patients are presented to the ED with traumatic lacerations every year, of which the most commonly occurring lacerations are usually located in the upper extremity (35%), followed by the face (10%), trunk (14.5%), lower extremity (12.5%) and head/neck (10%) [1]. These lacerations are usually treated to restore function through meticulous wound closure, prevent infections on the wound, and ensure a functional and aesthetically acceptable scar is achieved [2]. Previous research has shown that most wounds heal without complications, irrespective of the wound management therapy used. However, Quinn and colleagues reported that despite a low occurrence of wound infections (2.6%), improper wound management is likely to lead to increased wound infection, patient discomfort, and dissatisfaction [3].

Most of the traumatic lacerations presented to the ED are usually witnessed among children. These lacerations require wound closure which is painful and may result in one of the most unpleasant experiences for the children. Therefore, pain management is essential during wound repair. In fact, previous studies have shown that parents are willing to spend more time and money on “painless” treatment. Walsh and Bartfield reported that 65% of the parents were willing to spend an additional hour in the ED for their children to undergo “painless” treatment. Additionally, 77% of parents were willing to pay $15, while 37% considered paying as much as $100 for their children to undergo “painless” treatment [4].

Subcutaneous lidocaine injections remain the most effective anesthesia for most lacerations. However, the technique is usually painful and is less tolerated by children. Hence, non-invasive topical anesthetics are usually considered in pediatric wound management [2,5]. Topical anesthetics also have other advantages including the absence of wound margin distortion during repair, the provision of rapid and effective anesthesia for a reasonable time, and fewer side effects [6]. Some of the most commonly used topical anesthetics include a combination of tetracaine, adrenaline, and cocaine (TAC), a combination of lidocaine, epinephrine, and tetracaine (LET), and a eutectic mixture of local anesthetics (EMLA). EMLA cream is a combination of 2.5% lidocaine and 2.5% prilocaine suspended in an oil-in-water emulsion. This combination liquid is usually carried out at room temperature, allowing the formation of anesthetic emulsion droplets in the cream and the efficient transdermal spread of the active ingredients. Even though the EMLA is considered effective in pain management, it is also associated with several adverse events including blanching and redness at the application site [7]. On the other hand, LET is a topical anesthetic consisting of 4% lidocaine, 0.05% epinephrine, and 0.5% tetracaine which is usually applied directly to the wound before the skin repair procedure. Epinephrine is essential in causing localized vasoconstriction which improves the effectiveness of the local anesthetic and limits bleeding from the wound during repair [8]. Previous studies have also shown that it improves the procedural success rate and reduces procedural time as it decreases patient movement and pain [9].

Even though evidence shows that LET and EMLA are effective in wound management, very little research has been conducted on pediatric patients. Therefore, this systematic review was carried out to determine the efficacy of EMLA and LET for topical use in children during wound management.

## Review

Protocol and registration

This systematic review and meta-analysis followed the PROSPERO database protocol and registration. Consequently, the preparation of this article observed and followed the Cochrane Collaboration guidelines, and was reported as per the PRISMA (Preferred Reporting Items for Systematic Reviews and Meta-Analyses) guidelines.

Eligibility criteria

Relevant studies identified using the search strategy were scrutinized by two reviewers using the inclusion and exclusion criteria. The inclusion criteria for the present study were as follows; Accepted and published manuscripts written in English. This criterion was used to help us avoid direct translation of scientific terms, which could result in loss of meaning and context, Studies including a majority or complete participants less than 18 years, Studies that compared either EMLA or LET to other topical or local anesthetics in wound management, and Studies with more than 10 participants. This specification was vital since larger sample sizes would improve the statistical power of our meta-analyses.

On the other hand, the specified exclusion was as follows; accepted and published articles written in languages other than English, studies that included adult participants (>=18 years), articles that compared other topical anesthetics other than EMLA or LET, Articles that compared either EMLA or LET in painful procedures not related to wound management, articles which were designed as systematic reviews and meta-analyses, abstracts without evidence of full articles, letters to the editor or case reports, and articles conducted on animal subjects or human models.

Literature search

Using two methods, a detailed literature search was carried out per the PRISMA guidelines. First, a database search was performed on 5 electronic databases, including PubMed, Medline, Web of Science, Embase, and Google Scholar. This method involved a well-outlined search strategy consisting of specific keywords combined using the Boolean expressions “AND” and “OR.” The search strategy was as follows: (EMLA OR lidocaine-prilocaine gel) AND (LET OR lidocaine-epinephrine-tetracaine gel OR LAT OR lidocaine-adrenaline-tetracaine gel) AND (Topical anesthetics) AND (wound management OR wound repair OR wound closure OR laceration management) AND (children OR pediatrics OR infants). This search strategy was not limited to any specific period. The second method involved scrutinizing the reference lists of identified relevant articles for additional studies.

Data extraction

The extraction of relevant data from the included studies was carried out by two reviewers (H.A.Z. and H.F). These reviewers primarily collected the following data: Author ID (first author’s surname and publication year), participants’ characteristics, including sample size, mean age and gender distribution, type of wound being managed, interventions (experimental and control), and the main outcomes. The primary outcome of the present study was the visual analog pain Scale (VAS) which consists of a 10 cm line with two endpoints representing 0 (‘no pain’) and 10 (‘pain as bad as it could possibly be). The patient was asked to rate their current level of pain by placing a mark on the line. A ruler was used to measure the distance in centimeters from the ‘no pain marker’ (or zero) to the current pain mark. This provided a pain intensity score out of 10. For example, 6 out of 10 (or 6/10), while the secondary outcome was adverse reactions to the interventions. Any discrepancies in the recorded data were reconciled through consensus, and if this could not be achieved, a third reviewer was consulted.

Quality Assessment

**Figure 1:**
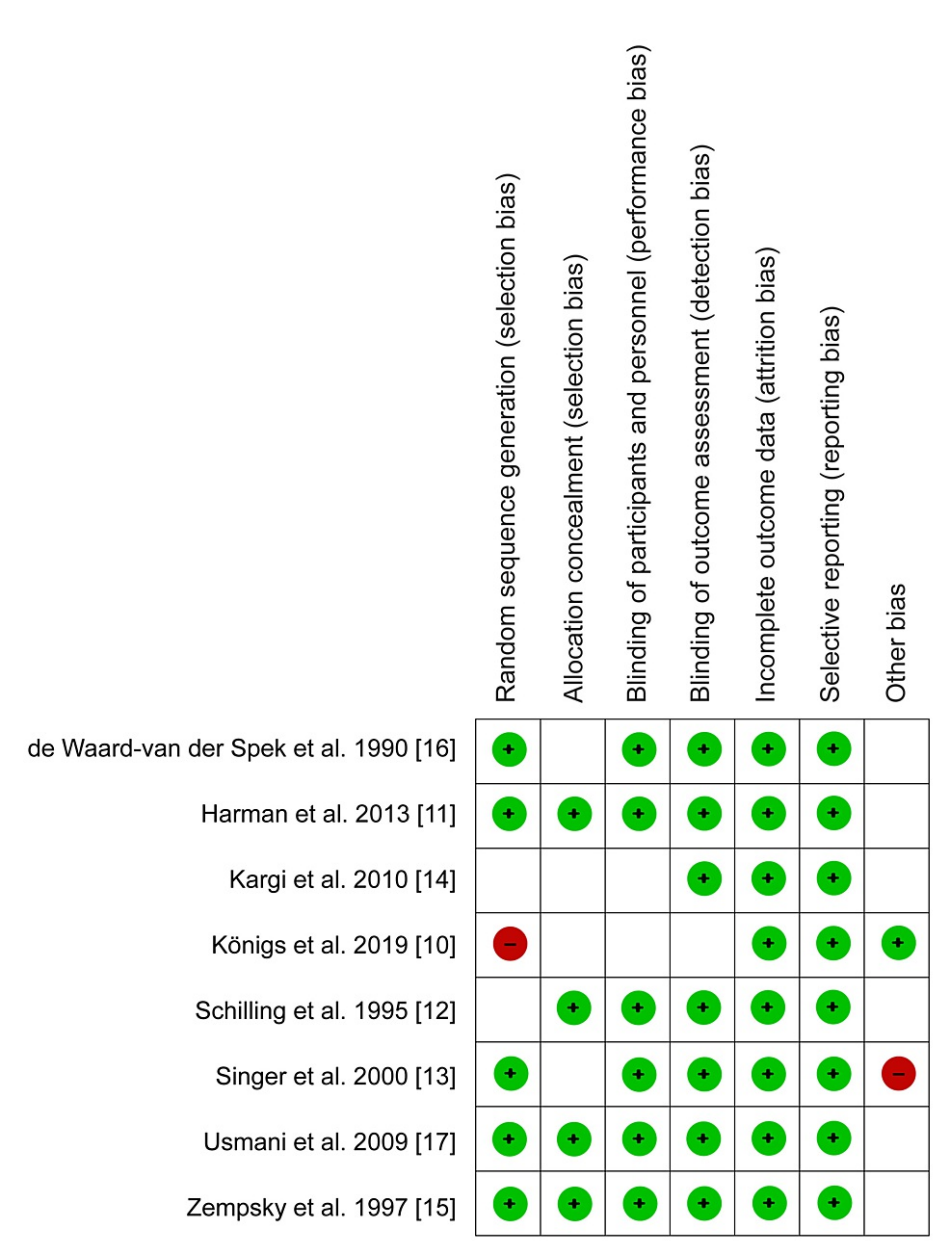
Risk of bias summary Königs et al.,2019 [10], Harman et al.,2013 [11], Schilling et al.,1995 [12], Singer et al.,2000 [13], Kargi et al.,2010 [14], Zempsky et al.,1997 [15], de Waard-van der Spek et al.,1990 [16], Usmani et al.,2009 [17]

**Figure 2:**
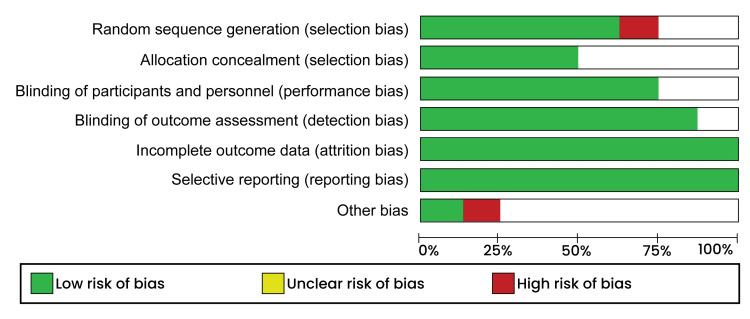
Risk of bias graph

The quality assessment of selected studies was individually conducted per the Cochrane handbook for systematic review criteria using the risk of bias tool provided in the Review Manager software (RevMan version 5.4.1). The assessment criteria were based on the following elements; selection, performance, attrition, and reporting bias, of which each element was categorized as either “low risk,” “high risk,” or “unclear risk.” A low risk of bias was used to explain a sufficiently addressed element, while a high risk of bias meant that a specific element was either insufficiently or not well addressed. On the other hand, situations, where the reviewers could not make any clear judgment on a specific element led to categorization as unclear risk of bias. The risk of bias summary is presented in (Figure 1) while the risk of bias graph is presented in Figure 2.

Synthesis of results

The pooled effects of all outcomes were carried out using RevMan v5.4.1. The consequences of pain scores were continuous; thus, their effect sizes were calculated using the Standard mean difference (SMD). On the other hand, results related to the number of adverse reactions were discrete; thus, their effect sizes were calculated using the Odds ratio (OR). During the analysis, a 95% CI was chosen, and the statistical significance was defined by p<0.05. A random effect model was also chosen to accommodate the expected heterogeneity due to varied sample sizes and pain score measures. The heterogeneity was measured using the I2 statistics, of which values ranging from 0 - 50%, 51 - 70%, and >70% were considered low, moderate, and high, respectively. All the results of our meta-analyses were presented using forest plots.

Results

Search Results/Study Selection

**Figure 3:**
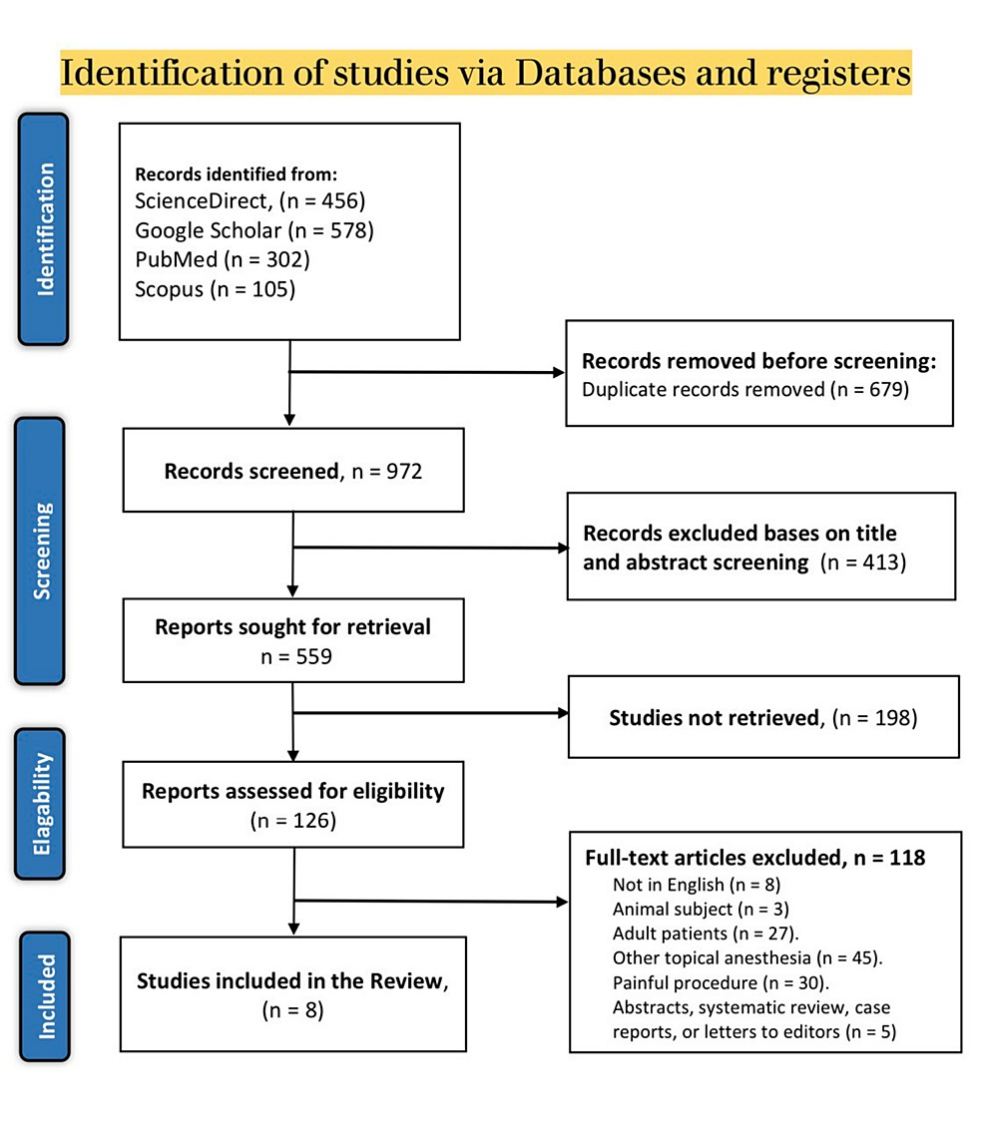
PRISMA flow diagram of the literature search results. PRISMA: Preferred Reporting Items for Systematic Reviews and Meta-Analyses.

A thorough search through the previously mentioned databases resulted in 1651 topic-related articles. These articles were screened for duplicates, and 679 duplicate articles were excluded. The remaining articles then had their titles and abstracts screened, of which 559 met the screening criteria. Of the 559 articles, 433 were not retrieved, and 126 were evaluated using the eligibility criteria for the present study. The evaluation led to the inclusion of only eight articles while the other articles were excluded as follows: 8 were published in different languages, three either included animal participants or human models, 45 evaluated other topical anesthetics, 27 included adult patients only, 30 either evaluated EMLA or LET on painful procedures that were unrelated to wound management and five were either designed as systematic reviews and meta-analyses, letters to the editors, case reports or abstracts without full articles. The selection results are shown using the PRISMA flow diagram (Figure 3).

Study Characteristics are presented in Table 1 below.

**Table 1:** Study Characteristics EMLA; Eutectic Mixture of Local Anaesthetics: TAC; tetracaine, adrenaline, and cocaine: LET; Lidocaine, Epinephrine, and Tetracaine: RCT; Randomized controlled trial: VAS; Visual analogue Scale, vs; Versus.

Author ID	Study design	Participants’ characteristics	Wound being managed	Experimental intervention	Control intervention	Main Outcomes
Königs et al.,2019 [10]	Prospective multicenter study	53 (aged 3 – 16 years).	Skin lacerations	37 patients were subjected to LET	22 subjected to EMLA with mepivacaine infiltration	Insignificant pain score differences were recorded after secondary injection (2.6 (2.0) vs. 4.0 (2.2), p = 0.20, for LET and EMLA groups, respectively). 3 of 37 patients in the LET group and 1 of 22 in the EMLA group experienced some complications. Significantly higher patient satisfaction after the procedure was observed in the LET group than EMLA group (LET 1.59 [0.60] vs. EMLA 2.04 [0.90], p ¼ 0.018).
Harman et al.,2013 [11]	RCT	203 (121 males and 82 females; aged 3 months to 17 years).	Minor lacerations on the face, torso, trunk or extremities	105 were subjected to LET	98 were in the placebo group	Based on color VAS scores, patients in the LET group showed significantly low pain than placebo (0.50 (0.25–1.50) vs. 1.00 (0.38–2.50), p=0.01, respectively). Based on the Face pain scale, patients in the LET group had significantly lower pain than placebo 0.00 (0.00–2.00) vs. 2.00 (0.00–4.00), p < 0.01, respectively).
Schilling et al.,1995 [12]	RCT	151 (100 male and 51 females; mean age 6.2+3.4 years)	Face or scalp lacerations.	73 were treated with TAC	78 were subjected to LET	No significant difference was observed in the meantime of effective anesthesia between the TAC and LET groups (19.18_+9.33 minutes vs. 19.23+10.81 minutes, p=0.98, respectively).
Singer et al.,2000 [13]	RCT	43 (13 females and 30 males; median age 13 years).	Face and extremities lacerations.	22 were subjected to LET	21 were in the placebo group.	The pain of injection was significantly lower among patients in the LET than in the placebo group (22 (10, 33) vs. 42 (20, 53) mm, p=0.02, respectively). No adverse events were recorded in either LET or placebo groups.
Kargi et al.,2010 [14]	RCT	30 (11 female and 19 males; mean age: 11.3 (8 – 15) years)	Face burn	15 were subjected to Lidocaine-prilocaine cream 5%.	15 were in the control group.	1 Patient in the experimental group showed moderate pain in the first 8-hour period, while 2 patients expressed moderate pain after the second 8-hour period. All wounds had healed in the second week, and no infectious, allergic or cardiovascular complications were recorded.
Zempsky et al.,1997 [15]	RCT	32 (aged 5 to 18 years)	Extremity wounds	16 were subjected to EMLA	16 were subjected to TAC	There was an insignificant difference in VAS pain scores between the EMLA and TAC groups (4.6+2.6 4.0+2.5, p= 0.50, respectively). 1 case of wound dehiscence was recorded in both groups, but no wound infections were recorded.
de Waard-van der Spek et al.,1990 [16]	RCT	83 (aged 4 – 12 years).	Moluscum contagiosum lesions	58 received EMLA cream	25 were subjected to a placebo cream	Ten patients in the 60-minute EMLA group were observed to have redness, while five patients in the 30-minute EMLA group had redness. Only two patients in the placebo group had redness.
Usmani et al.,2009 [17]	RCT	90 (88 males and two females; aged 4 – 12 years)	Wounds after inguinal hernia repair	30 received EMLA cream	30 were subjected to lidocaine, and 30 were in the control group	Time to discharge from the recovery room was shorter in patients in the EMLA and lidocaine group than in the control group (67 + 14 vs. 66 + 14 + 72 + 17, respectively). A significantly lower number of patients requiring rescue analgesics was observed in the study groups than in the control group (5 vs. six vs. 20 for EMLA, lidocaine, and control, respectively).

Pain Reduction

**Figure 4:**
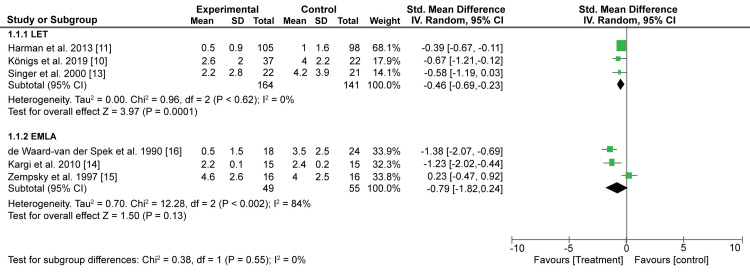
A forest plot showing the effect of LET and EMLA on VAS pain scores LET; Lidocaine, Epinephrine, Tetracaine, EMLA; Eutectic Mixture of Local Anesthetics, VAS; Visual analogue scale. Königs et al.,2019 [10], Harman et al.,2013 [11], Singer et al.,2000 [13], Kargi et al.,2010 [14], Zempsky et al.,1997 [15], de Waard-van der Spek et al.,1990 [16]

Pain measurement was done using the Visual analog scale in most studies (VAS). For the uniform meta-analysis, 100-mm VAS point scores were converted to 10-cm VAS point scores. Pooled results from 3 studies showed that LET significantly reduced the pain witnessed by children during the wound management than the control (SMD: -0.46; 95% CI: -0.69, -0.23: p < 0.0001) (Figure 4). On the other hand, EMLA reduced the pain recorded in children during wound management than control interventions. However, the difference did not reach a statistical significance (SMD: -0.79; 95% CI: -1.82, -0.24: p = 0.13) (Figure 4).

Adverse Reactions

**Figure 5:**
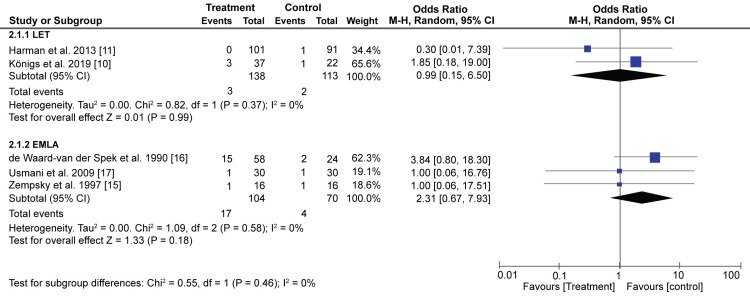
A forest plot showing the number of adverse reactions related to LET and EMLA LET; Lidocaine, Epinephrine, and Tetracaine, EMLA; Eutectic Mixture of Local Anesthetics. Königs et al.,2019 [10], Harman et al.,2013 [11], Zempsky et al.,1997 [15], de Waard-van der Spek et al.,1990 [16], Usmani et al.,2009 [17]

The number of adverse reactions associated with the topical anesthetics was minimal (3/138 for LET and 17/104 for EMLA). Pooled results from 2 studies show that an insignificant difference was observed in terms of adverse reactions between the LET and control groups (OR: 0.99; 95% CI: 0.15, 6.50; p = 0.99) (Figure 5). Similarly, the results of our meta-analysis show that there was no significant difference in the adverse reactions observed in the EMLA treatment group and the control group (OR: 2.31; 95% CI: 0.67, 7.93; p = 0.18) (Figure 5).

Discussion

Pain is common during the management of wounds in patients of all ages. Therefore, pain management is vital for successful procedures, especially in children during wound repair. Our meta-analyses showed that when EMLA and LET were topically applied for pediatric patients had a reduction in pain. However, LET showed a significant pain reduction compared to control interventions, while EMLA showed an insignificant difference compared to control interventions.

The significant pain reduction recorded in the present study for LET application is consistent with previous literature, which included patients of all ages. Ernst et al. [18] reported that for patients undergoing extremity, face, and scalp lacerations repair, the LAT gel application showed significantly lower patient-reported VAS than the injectable lidocaine (0 (0 - 0.15) vs. 1.2 (0.15 - 2.75); p = 0.001, respectively). However, the results of that study also showed that the pain witnessed during suturing was insignificantly different for patients in the LAT and injectable lidocaine groups (0 (0 - 1.35) vs. 0(0 - 0.6); p = 0.48, respectively). Similarly, a randomized study of patients with head, trunk, and limb wounds reported that significantly less pain was recorded among patients in the LAT group during application than lignocaine infiltration group (1.50 (0.40) vs. 3.5 (0.46), p<0.01, respectively). However, the pain scores recorded during the wound repair showed an insignificant difference (2.5 (0.52) vs. 2.6 (0.58); p = 0.87 for LAT and lignocaine infiltration groups, respectively) [19]. Another previous research work by Ernst and colleagues showed that the patient-recorded VAS pain scores were insignificantly different between the LAT and TAC groups (0 (0 - 4) vs. 0 (0 - 6); p = 0.266, respectively)[20]. The study also showed that based on the physician-ranked VAS scores, LAT was more effective in pain reduction than TAC (0 (0 - 5) vs. 1 (0 - 6); p = 0.0093, respectively).

Apart from pain reduction, patient satisfaction has also been used to evaluate the efficacy of LET. Königs et al. [10] reported that overall satisfaction was excellent for children undergoing skin repair in both LET and EMLA groups. However, the directly measured patient satisfaction after the skin repair procedure was significantly higher among patients that received LET than EMLA (1.59 [0.60] vs. 2.04 [0.90], p = 0.018, respectively). Similarly, a previous study that evaluated the effectiveness of LAT gel among children with finger lacerations reported that for patients where LAT gel was successful, 94.3% of patients and 93.9% of parents were willing to use the LAT gel again. Even for patients in whom the LAT gel was unsuccessful, responses for willingness to use the LAT gel again were relatively good (44.4% and 50% for parents and patients, respectively) [21].

Cases of additional anesthesia, especially after the procedures, have also been reported. A previous randomized study by Adler et al. [22] showed that LET gel significantly reduced the number of patients requiring infiltrative analgesia. In that study, out of the 30 patients receiving the LET gel, only 13 (43.3%) required additional anesthesia. Similarly, Vandamme and colleagues reported that of the 89 patients treated with LAT gel, only 21 (23.6%) were in need of additional anesthesia [23]. The difference between these two studies can be attributed to the fact that the wounds were located in different parts of the body. Even though additional anesthesia may be required by patients receiving LET gel, a randomized trial by Singer and colleagues shows that the infiltration is usually less painful and more adequately anesthetized compared to wounds that have not been applied with the LET gel [13].

Additionally, LET has been found effective in the reduction of the length of ED stay by reducing the treatment time for pediatric patients undergoing laceration repairs. Schilling et al. [12] compared the efficacy of TAC and LET among children undergoing laceration repair and found that for patients that achieved complete anesthesia, the mean effective anesthesia was comparatively similar in the TAC and LET group (19.18+9.33 vs. 19.23+10.81, p = 0.98, respectively). Similarly, a study by Priestley and colleagues showed that LET at triage reduced the treatment time for patients with minor lacerations by about 30 minutes. The results of that study showed that the mean treatment time between the LET and other treatment groups was 107 minutes and 138 minutes, respectively (effect size 31 minutes; 95% CI 15 to 71 minutes) [24]. Combining this outcome with the fact that LET significantly reduces pain for pediatric patients undergoing wound management, it is possible that LET can be an effective, painless therapy for children. Additionally, the epinephrine in the LET has vasoconstriction properties which help reduce bleeding during wound management. This property can be seen in the study by Harman et al. [11] which showed that a significantly higher number of patients in the LET group had complete wound hemostasis (no bleeding) compared to the placebo (79 (78.2) vs. 54 (59.3); p = 0.008).

It is also vital to note that various factors can influence the efficacy of LET among pediatric patients. The first influencing factor is the location of the wounds. Vandamme and colleagues reported that the VAS scores during needle probing were significantly lower for patients with head lacerations compared with extremities/trunk and fingers/toes lacerations (0 (0.0 - 2.0) vs. 2.0 (0.5 - 3.0) vs. 3.0 (2.0 - 4.0); p = 0.001, respectively) [23]. Similarly, the study showed that head wounds required less additional analgesia compared with extremities/ trunk and finger/toes. The results obtained in this study can be explained by the fact that the face and scalp have relatively high vascularity of lacerations leading to better absorption of the LET gel [25]. In addition, the lacerations on the head are usually smaller in size compared to other areas and, thus, may require less anesthesia. The length of the wound also seems to affect the efficacy of LET. It seems as if LET has much more effect on shorter lacerations as compared to longer lacerations. In a study by Königs et al. [10] where the average length of lacerations was 3.31 (1.97) cm, and VAS pain scores of 2.6 (2.0) were recorded in the LET group. On the other hand, a study by Harman et al. [11] where the average laceration length was 1.15 ± 0.60 cm, and the achieved VAS score was 0.50 (0.25-1.50). Despite this deduction, it is important that future trials compare the effect of lacerations length on the efficacy of LET. Evidence from previous studies also shows that LET in gel and solution form may affect efficacy. Resch et al. [26] compared the efficacy of LET solution and gel in 200 children with face and scalp lacerations and found that more patients in the gel group had achieved complete anesthesia compared to the solution group (64 (85%) vs. 66 (76%), respectively). However, the study shows that the time to achieve an anesthetic effect between the two formulations was the same (21 minutes). The study also claimed that the gel was easier to use as opposed to the solution. The easy use of gel can be explained by the fact that in solution form, LET tends to run out of the wound and can contaminate the mucous membrane or ocular structures. On the other hand, the gel tends to remain in place and keeps the anesthetic agents better contained.

Despite the evidence showing that LET is effective during wound management, it is subject to adverse reactions. However, the number of adverse reactions recorded in our meta-analysis is very small. These results are supplemented by a previous research study by Lee et al. [19], which also recorded a very small number of complications (7/25). According to that study, the number of wound infections wound dehiscence and lost stitches in the LAT gel group were 5, 1, and 1, respectively. Similarly, a study by Ernst et al. [20] reported that neither of the patients in the LAT gel group had any complications, but 2 cases of wound infection and hematoma were recorded in the TAC group. Most of the studies included in our systematic review also reported that there were zero cases of complications observed in the LET or control groups. This shows that LET can achieve painless wound management with fewer complications making it an effective therapy among children.

EMLA also reduced pain in pediatric patients during wound management; however, a comparison with control interventions shows the difference is insignificant. These results are supported by previous research works that included children and adult patients. Singer et al. [27] after evaluating the effect of topical anesthetics among 60 patients aged 1 to 59 years with facial lacerations, found that there was no significant difference in VAS pain scores between the EMLA and LET group for children aged less than eight years (7 (1-29) vs. 14 (5-32) mm; p = 0.46). However, other studies have shown some contradicting information. Park and colleagues evaluated the efficacy of topical anesthesia on facial lacerations for patients aged >=16 years and found that pretreatment with EMLA had a significant reduction in pain scores than patients that underwent the routine process (2.4 vs. 4.5 on VAS, P<0.05, respectively) [28]. A recent study evaluating the efficacy of EMLA in 60 patients undergoing dressing of painful chronic leg ulcers also reported that the EMLA group had significantly lower VAS pain scores than the control group after a 4-week intervention period (3.39 (2.16) vs. 4.82 (2.27); p = 0.04, respectively)[29]. When the intervention period was extended to 12 weeks, an insignificant difference was recorded (3.25 (2.18) vs. 4.29(2.30); p = 0.08, for the intervention and control groups, respectively).

The need for additional anesthetic is also regarded as an effective measure for the efficacy of EMLA during pediatric wound management. A study that compared the efficacy of EMLA to lidocaine infiltration in children that had wounds after inguinal hernia repair reported that the patients in the control group had a significantly shorter time to rescue anesthesia than the EMLA group (71 + 68 mins vs. 143 + 84 mins, p < 0.05, respectively) [17]. The study also showed that the number of people requiring fentanyl as rescue analgesia was significantly lower in the EMLA group (5 (17%) vs. 6 (20%) vs. 20 (67%), for EMLA, lidocaine infiltration and control group, respectively). Kargi and colleagues evaluated the need for additional analgesia for children undergoing postburn treatment, and their results showed that at the 24 hours follow-up 9 cases of additional analgesia were recorded in the EMLA group (1 in the first 8 hours, 3 in the second 8-hour, and 5 in the third hour) [14]. Comparing these results to the control group were able to deduce that patients in the control group recorded the highest number of patients requiring additional anesthesia (21 cases). Zempsky and Karasic [15] also showed that patients in the EMLA group often required less additional anesthesia compared with the TAC group. Previous research also shows that EMLA has improved patient satisfaction among children and adult patients [28].

Even though our research did not include studies about topical anesthesia on procedural pain, extensive research has shown that EMLA effectively provides anesthesia for children undergoing painful procedures such as venous cannulation, circumcision, port-a-Cath puncture, and caudal block. Hopkins et al. [30] used a 5% EMLA cream for children aged 1 - 5 years to undergo venipuncture and found that the patients experienced less pain when subjected to the procedure. However, when the application time was adjusted from 30 to 300 minutes, no significant variations in the mean pain scores. Similarly, Manner and colleagues, after evaluating the effectiveness of EMLA cream in children subjected to venous cannulation, showed that patients in the EMLA group had significantly lower VAS pain scores than the placebo (p < 0.001) and control group (p <0.01) [31]. Another study compared the efficacy of EMLA cream to dorsal penile nerve block in alleviating pain in children undergoing circumcision [32]. That study concluded that EMLA is an effective and safer method of providing anesthesia during circumcision in children since it helps to avoid the rare but serious complications associated with a dorsal penile nerve block and is simpler to perform. Other related studies have reported that EMLA may be more effective than a placebo in alleviating pain during circumcision, but it is less effective than the dorsal penile nerve block [33-35]. A randomized trial by Bishai et al. [36] also compared the efficacy of EMLA to amethocaine gel in pediatric patients subjected to port-a-Cath puncture and found that EMLA was effective in reducing pain associated with the procedure. However, the comparison showed that the difference was statistically insignificant (2.0 (1.4) vs. 1.5 (1.5); p = 0.09, for amethocaine gel and EMLA cream, respectively).

It is also important to note that the application time of EMLA may be disadvantageous for emergencies. Results by Usmani showed that the application time required for children undergoing hernia repair was 2 hours [17]. Similarly, Zempsky and Karasic reported that despite EMLA being applied directly to the open wound, it took at least 1 hour for sufficient anesthesia to be observed [15]. However, this does not mean that EMLA cannot have a shorter application time. Singer and colleagues showed that EMLA had almost similar application time to LET during wound management (40 (34-50) vs. 34 (26-45) minutes, p = 0.21). EMLA is also subject to various complications. Usmani reported that 1 case of subcutaneous infection at the incision site was recorded in the EMLA group after 10 - 15 days. However, the infection was effectively treated using fusidic acid ointment, which was applied for 4 - 5 days. A randomized trial evaluating the efficacy of EMLA in extremity wounds reported that despite not having any wound infections, 1 case of wound dehiscence before the removal of sutures was recorded in the EMLA group. De Waard-van der Spek et al. [16] also reported cases of wound redness. According to the results of that study, the number of wound redness cases increased with the EMLA application time (0% (0/22) vs. 28% (5/18) vs. 56% (10/18) for 15-minutes, 30-minutes and 60-minutes EMLA application).

Limitations

The current study was subject to numerous limitations, including high heterogeneity in the EMLA pain score analysis. This heterogeneity can be attributed to the inclusion of patients with varying wound types. Similarly, the varying control interventions used in each study could have contributed to the increased heterogeneity. Based on this heterogeneity, the results should be carefully interpreted. However, the studies used in the present study were majorly randomized trials with good methodology, thus eliminating publication bias. Additionally, the study was based on children, but the lack of sufficient literature led to the inclusion of studies where most patients were children. This might have input some bias in our meta-analysis results. The eligibility criteria also ensured that studies published in languages other than English were not retrieved. With this specification, we could have omitted relevant studies that could have otherwise informed our scientific research and improved the statistical power of our meta-analysis. Some of the included studies relied on patient information regarding infections which may have introduced bias on the number of complications since some patients may not have identified infections and could not be accounted for in the final results.

## Conclusions

Our study reveals that the topical application of EMLA and LET on pediatric wounds is effective. LET showed that it significantly reduced pain than control interventions; however, EMLA had an insignificant pain reduction compared to control interventions. These results imply that EMLA and LET can be used effectively for pain-free wound repairs in pediatric patients; however, focused research protocols are needed to directly compare the topical anesthetic to demonstrate which topical anesthetic is more appropriate for children. Comparing the number of recorded adverse reactions, we can hypothesize that more complications are observed when using EMLA. However, future research should be conducted to show which topical anesthetic is more susceptible to adverse reactions. Furthermore, evidence from the literature has also demonstrated that EMLA may be disregarded for emergencies due to its longer application time. However, further investigations are required to compare it to LET.
